# Response of Medical Cannabis to Magnesium (Mg) Supply at the Vegetative Growth Phase

**DOI:** 10.3390/plants12142676

**Published:** 2023-07-18

**Authors:** Dalit Morad, Nirit Bernstein

**Affiliations:** 1 Institute of Soil Water and Environmental Sciences, Volcani Center, 68 HaMaccabim Road, P.O. Box 15159, Rishon LeZion 7505101, Israel; 2 The Robert H. Smith Faculty of Agriculture, The Hebrew University of Jerusalem, Rehovot, Israel

**Keywords:** cannabis, cannabinoid, development, magnesium, Mg, medical, root, vegetative

## Abstract

Recent studies demonstrated a significant impact of some major macronutrients on function and production of medical cannabis plants, yet information on the effect of most nutrients, including Mg, is scarce. Magnesium is required for major physiological functions and metabolic processes in plants, and in the present study we studied the effects of five Mg treatments (2, 20, 35, 70, and 140 mg L^−1^ Mg), on plant development and function, and distribution of minerals in drug-type (medical) cannabis plants, at the vegetative growth phase. The plants were cultivated in pots under controlled environment conditions. The results demonstrate that plant development is optimal under Mg supply of 35–70 mg L^−1^ (ppm), and impaired under lower Mg input of 2–20 mg L^−1^. Two mg L^−1^ Mg resulted in visual deficiency symptoms, shorter plants, reduced photosynthesis rate, transpiration rate, photosynthetic pigments and stomatal conduction in young-mature leaves, and a 28% reduction of total plant biomass compared to the optimal supply of 35 mg L^−1^ Mg. The highest supply level of 140 mg L^−1^ Mg induced a small decrease in physiological function, which did not affect morphological development and biomass accumulation. The low-deficient Mg supply of 2 mg L^−1^ Mg stimulated Mg uptake and accumulation of N, P, K, Ca, Mn, and Zn in the plant. Increased Mg supply impaired uptake of Ca and K and their root-to-shoot translocation, demonstrating competitive cation inhibition. Mg-deficiency symptoms developed first in old leaves (at 2 mg L^−1^ Mg) and progressed towards young-mature leaves, demonstrating ability for Mg in-planta storage and remobilization. Mg toxicity symptoms appeared in old leaves from the bottom of the plants, under 140 mg L^−1^ Mg. Taken together, the findings suggest 35–70 mg L^−1^ Mg as the optimal concentration range for cannabis plant development and function at the vegetative growth phase.

## 1. Introduction

Cannabis (*Cannabis sativa* L.) is among the earliest plants cultivated by mankind that has been grown for over 5000 years for diverse applications, including medicine, fiber production, and recreation [1,2,3]. Legal restrictions in the last decades limited research of the cannabis plant and its medical applications [3], and only recently, changes in regulations world-wide opened opportunities to close the knowledge gaps that are required to support the booming cannabis industry.

Cannabis is an annual short-day plant, and its production for the medical and recreational markets is based on development of inflorescences at the generative growth phase under a short photoperiod [4]. To ensure genetic uniformity for enhancing the chemical standardization of the plant product supplied to consumers, the plants are propagated vegetatively by rooted cuttings [5,6]. Vegetative development of the plant body under long photoperiod supports the development of inflorescences, which are the commercial yield, under short day (Shiponi and Bernstein, [7]). At the vegetative growth phase, the main stem of the cannabis plant produces alternate lateral branches that further branch to produce second- and third order branches. The transition to a short photoperiod induces a developmental shift in the plant body, and the apical meristems of the main stem and side branches, as well as lateral buds, develop inflorescences [5,8]. Therefore, plant structure, size, and function at the vegetative growth phase, prior to the transition to the short photoperiod, has a profound effect on the potential for yield production. Thereby, information on the cannabis plant morpho-physiology responses at the vegetative growth phase is vital for directing cultivation practices towards optimal production.

Abiotic factors, including mineral nutrition, have profound effects on plant development and function [9]. Mineral input, and its effect on the nutritional status of the plant, are of the main factors affecting vegetative development and architecture of the plant body [10,11], and hence the potential for reproductive growth [12]. We have previously demonstrated sensitivity of the cannabis plant to mineral supply at the vegetative and the reproductive growth phases: Optimum supply rates for medical cannabis were found to be 175 mg L^−1^ K [13], 30 mg L^−1^ P [8], and 160 mg L^−1^ N [14] for the vegetative phase; and 60 mg L^−1^ K [15], 30–90 mg L^−1^ P depending on the genotype [7], and 160 mg L^−1^ N [16] for the reproductive phase; and interaction between supply of individual macronutrients was demonstrated [17,18]. Information on the response of drug-type cannabis to other macro and micronutrients, including magnesium (Mg) is not available today.

Magnesium is an essential macronutrient for plant growth and metabolism [19,20]. It takes part in key physiological functions and metabolic processes of the plant, which may be disrupted under Mg deficiency or toxicity conditions. It involves in photosynthesis by activation of ribulose-1,5-bisphosphate carboxylase-oxygenase, which is a key enzyme in the photosynthesis process and the most abundant enzyme on earth [21], and as a central component of the chlorophyll molecule [22]. It has a key role in energy utilization in the plant by serving as a cofactor for many enzymes including adenosine triphosphate (ATP), the main source of energy in cells [23,24,25]. It is also essential for the synthesis of nucleic acids and proteins [10]; for regulation of carbohydrate metabolism [26]; and for the transport of assimilates from source to sink organs due to its involvement in phloem loading. Therefore, Mg deficiency disturbs the partitioning of assimilates in the plant, resulting in increased accumulation of assimilates in source leaves and reduced growth rate of sink organs, including roots [27]. Generally, Mg deficiency results in shorter roots, smaller shoots, necrotic spots on leaves, and interveinal chlorosis [22,28,29].

Magnesium is known to interact with other cations such as K and Ca for root uptake. High concentrations of these cations in the root zone may reduce Mg absorption to the plant and accumulation in the plant organs [30,31,32], and a high Mg supply can restrict root uptake of other cations [30]. The resulting changes to the tissue ionome can affect plant growth, function, and production. In cannabis, at the vegetative growth stage, Mg concentration in the plant organs decreases with the increase in K supply [13], and increases with the increase in P [8] and N availability [14], up to the optimal P and N concentrations.

The lack of information pertaining to the response of cannabis plants to Mg nutrition limits the development of optimal fertigation practices. The present study thereby focused on Mg nutrition of drug-type medical cannabis plants at the vegetative growth phase. The hypothesis guiding the workplan was that Mg nutrition affects the plants’ morpho-physiology and elicits developmental changes associated with changes to the plant ionome. To test this hypothesis, we studied physiological and developmental responses of the plants to Mg inputs ranging from 2 to 140 mg L^−1^ and conducted a chemical profiling of the plant organ’s ionome. The Mg range selected for the study was aimed at identifying optimal supply rates for Mg by encompassing supply inputs ranging from Mg deficiency to toxicity. Understanding the factors that affect the cannabis plant function and development at the vegetative growth stage is vital for achieving optimal plant architecture which is required for excelled inflorescence yield development at the generative growth phase.

## 2. Materials and Methods

### 2.1. Plant Material and Growing Conditions

The medical drug-type cannabis (*Cannabis sativa* L.) cultivar ‘Anafurna’ (Canndoc Ltd., Herzliya, Israel), was used as the model plant for the study. This cultivar has an indica-like morphology, and it contains similar concentrations of Δ^9^-tetrahydrocannabinol (THC) and cannabidiol (CBD) (~7%). The plants were propagated by cloning, from a single mother plant in vermiculite (Hydroisrael, Rishon LeZion, Israel). Fourteen-day-old rooted cuttings, selected for uniformity, were planted in 3 L plastic pots in a perlite cultivation medium (2-1-2, Agrekal, Habonim, Israel) and cultivated under a long-photoperiod for one week adjustment prior to the initiation of the treatments under the same growing conditions applied during the experiment. For this week and throughout the experiment, the plants were grown under long photoperiod (18/6 h light/dark), at 25 °C, in a controlled growing room, and irradiated with 400 μmol m^−2^ s^−1^ (Metal Halide bulbs; Solis Tek Inc., Carson, CA, USA; 25.9 mol m^−2^ d^−1^). Irrigation was supplied daily via 1 L h^−1^ discharge-regulated drippers (Netafim, Tel-Aviv, Israel), 1 dripper per pot. The volume of irrigation in each irrigation event was 330–500 mL/pot/day, set to allow 30% of drainage. Following the week of adjustment, the plants were randomly separated into 5 treatments of increasing Mg concentrations: 2, 20, 35, 70, and 140 mg L^−1^ (ppm), (e.g., 0.08, 0.82, 1.44, 2.88, and 5.76 mM Mg, respectively); 5 plants per treatment. The plants were cultivated under the differential Mg treatments for 30 days, until the end of the study. Routine chemical analyses of the irrigation solutions and the leachates confirmed that the concentrations of Mg in the irrigation solutions were consistent with the target concentrations for the treatments. The irrigation solution also contained (in mg L^−1^): 160 N (146 N-NO_3_^−^,14 N-NH_4_^+^), 30 P and 175 K, which are the optimal concentrations we identified in previous studies for the vegetative growth phase of medical cannabis [8,13,14], with 110 Ca, 1.7 Fe, 0.8 Mn, 0.4 Zn, 0.10 B, 0.04 Cu, 0.03 Mo, and 85 S-SO_4_ in the 4 lower Mg treatments and 144 S-SO_4_^−2^ in the highest Mg treatment. SO_4_ was selected as the accompanying ion for part of the highest Mg treatment since previous studies in the lab found that S uptake by cannabis plants does not increase with increased supply at the applied concentration range. The fertigation solutions were made with MAP (Mono-ammonium phosphate), potassium nitrate, calcium nitrate, magnesium sulphate, magnesium nitrate, sulphuric acid and MKP (mono-potassium phosphate). Micronutrients were supplied chelated with EDTA (Cu, Mn, Mo, Zn), EDDHSA (Fe) (Barkoret, ICL, Haifa, Israel), and B was supplied as B-7000 (ICL, Tel-Aviv, Israel). pH of the fertigation solutions was adjusted to 5.8 with NaOH. The experiment was conducted in a complete randomized experimental design, with 5 replicated plants per treatment. All measurements were conducted with 5 independent replications, following the experimental design. 

### 2.2. Inorganic Mineral Analysis

Concentrations of mineral nutrients in the plant organs were analyzed at the termination of the experiment, 4 weeks after the initiation of the Mg treatments. The plants were dissected into leaves, roots, and stems. Leaves and stems were rinsed twice with distilled water and blotted dry, and following removal from the growing media, the roots were gently rinsed three times in distilled water and blotted dry. Fresh weights of the plant organs were measured with a digital balance (Precisa 40SM-200A, Zurich, Switzerland). Dry biomass was measured after drying at 64 °C for 48 h. For the analysis, the dry tissue was ground to a powder and the samples were analyzed for concentrations of N, P, K, Ca, Mg, Fe, Mn, and Zn. Two different procedures were used for extraction of the elements from the plant tissue. For the analysis of Mg, Mn, Ca, Zn, and Fe, the ground tissue was heat-digested with the acids HNO_3_ (65%) and HClO_4_ (70%), and the minerals were analyzed with an atomic absorption-emission spectrophotometer (AAnalyst 400 AA Spectrometer, PerkinElmer, MA, USA). For the analysis of N, P, and K, the ground tissue was acid heat-digested with H_2_SO_4_ (98%) and H_2_O_2_ (70%) [33]. N and P were analyzed by an autoanalyzer (Lachat Instruments, Milwaukee, WI, USA), and K was analyzed by flame photometry (410 Flame Photometer Range, Sherwood Scientific Ltd., Cambridge, The Paddocks, UK).

For the evaluation of the effect of Mg on mineral uptake and root-to-shoot translocation, two factors were calculated, following Shiponi and Bernstein [8]: a bioaccumulation coefficient (BC) (L Kg^−1^) (Equation (1)), and a translocation factor (TF) (Equation (2)):

(1) $$BC=\frac{Concentration\;of\;the\;mineral\;in\;the\;plant}{Concentration\;of\;the\;mineral\;in\;the\;solution}$$

(2) $$TF=\frac{Concentration\;of\;the\;mineral\;in\;the\;shoot}{Concentration\;of\;the\;mineral\;in\;root}$$

### 2.3. Determination of the Osmotic Potential, Relative Water Content, and Membrane Leakage

The plants were sampled for osmotic potential, membrane leakage, and relative water content determination 27 days after the initiation of the fertilization treatments, i.e., 33 days after planting (3 days before the destructive harvest).

Osmotic potential as an indicator of water relations of the plant tissue, and membrane leakage (leakage of ions from the tissue) as an indicator of stress level of the tissue were analyzed in fan leaves at two developmental stages. The leaves analyzed were the youngest mature leaf on the main stem (young-mature leaf), and an older-mature leaf from the fifth node from the top of the main stem. Following dissection from the plant, both leaves were washed twice with distilled water and gently dried. For the **osmotic potential** measurements, two leaflets from each fan leaf were immediately frozen in −80 °C in a 1.7 mL Eppendorf test tube, and the analysis was conducted, as is described by Saloner and Bernstein [14]. In short, partially thawed samples were crushed inside the tube, the cell sap was extracted by centrifugation at 6000 rpm and 4 °C for 5 min, and 50 µL of the extracted cell sap was used for osmotic potential analysis with a micro-osmometer (Osmomat 3000, Gonotec, Berlin, Germany). For **membrane leakage** determination, the middle leaflet was put into a 50 mL test tube filled with 30 mL of distilled water and shaken for 24 h, and the EC of the leaf solution was measured. The samples were then autoclaved for 30 min, and the EC of the solution was measured again after the samples reached room temperature. Membrane leakage was calculated as the ratio of the EC solution before and after disruption of the cell membranes by autoclaving, and is presented as percentage (%). For **relative water content** (RWC) analysis, the second youngest mature fan leaf on the main stem was weighed immediately following dissection from the plant and placed in a test tube containing 50 mL of distilled water. Following soaking for 24 h, the leaves were weighed again. Dry weight was measured after desiccation at 64 °C for 48 h. RWC was calculated following [34].

### 2.4. Photosynthetic Pigments and Gas-Exchange Parameters

Transpiration rate, net photosynthesis rate, stomatal conductance, and intercellular CO_2_ concentration were measured as well on the youngest mature fan leaf on the main stem, and on an older-mature leaf from the fifth node from the top of the main stem. The measurements were conducted with a Licor system (6400 XT, LI-COR, Lincoln, NE, USA), 26 days after the initiation of the Mg treatments (4 days before the destructive harvest). Intrinsic water use efficiency (WUEi), which is the response of WUE at the leaf level was calculated by dividing the net photosynthesis results by the stomatal conductance. The measurements were conducted for five replicated plants per treatment. For photosynthetic pigments analysis, 5 leaf discs, 6 mm in diameter were sampled from the central leaflet of the two designated leaves, immersed in 0.8 mL ethanol (80%), and kept at −20 °C until analysis. The extraction of the pigments from the tissue, pigment analysis, and calculations of chlorophyll a, b and, carotenoids were conducted following Shiponi and Bernstein [7] and Lichtenthaler and Welburn [35].

### 2.5. Plant Architecture and Development

The stem diameter and the number of nodes on the main stem were measured at the termination of the experiment, on day 30 from the beginning of the Mg treatments. Stem diameter was measured at the location 3 cm from the plant base with an electronic caliper (YT-7201, Signet Tool International Co., Ltd., Shengang District, Taiwan). Plant height was measured 5 times throughout the experiment as the distance from the plant base to the top of the main stem. The measurements were conducted on five replicated plants per treatment. Biomass of the leaves, stems, and roots was measured at the termination of the experiment, 30 days after the initiation of the Mg treatments. Dry weights were recorded following desiccation at 64 °C for 72 h.

### 2.6. Statistical Analyses

The data were analyzed by two-way or one-way analysis of variance (ANOVA) followed by Tukey’s HSD post-hoc test for separation of means. The data met the assumptions of normality and homogeneity of variances. The analysis was performed with Jump software (Jump package, version 9, SAS 2015, Cary, NC, USA).

## 3. Results

### 3.1. Plant Growth and Development and Visual Appearance

**Figure 1:**
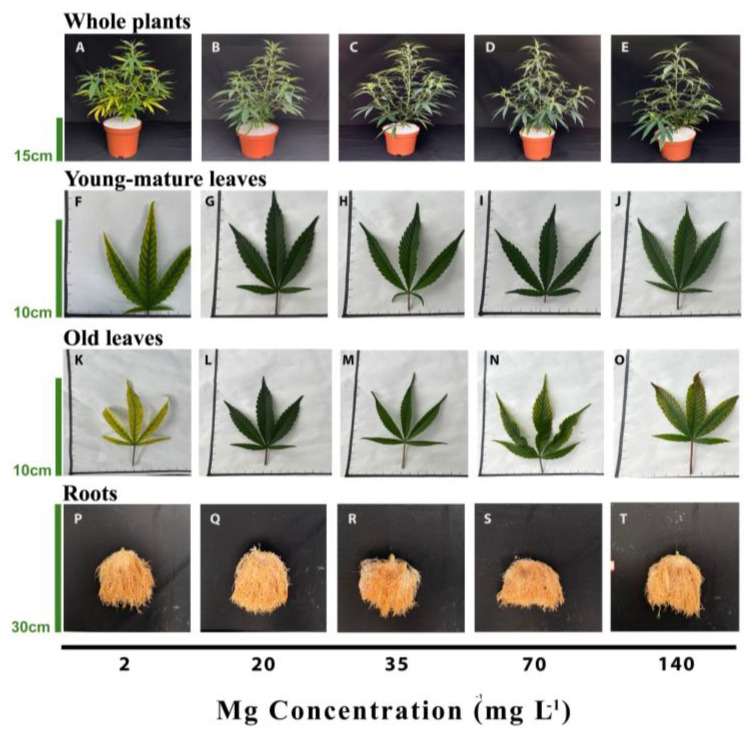
Visual appearance of the plants (top row, **A**–**E**), the youngest-mature leaves (second row, **F**–**J**), old leaves from the bottom of the plant (third row, **K**–**O**) and roots (bottom row, **P**–**T**) under 2, 20, 35, 70, and 140 mg L^−1^ Mg supply. Images were taken 30 days after the initiation of the Mg treatments.

The visual appearance of the shoot, young leaves, mature leaves, and roots reflects the plant response to the level of Mg supplied (Figure 1). The plants supplied with 2 mg L^−1^ Mg showed interveinal chlorosis. Such symptoms were apparent in both the young-mature and old-mature leaves at the termination of the experiment and were more severe in young-mature leaves (Figure 1A,F,K). Plants provided with higher Mg concentrations (20–140 mg L^−1^) appeared similar and unharmed, except that under the high concentration treatments of 70 and 140 mg L^−1^ Mg, symptoms of toxicity, e.g., necrotic spots, were apparent on old leaves from the bottom of the main stem (Figure 1N,O). There was no difference in the appearance of the roots between treatments (Figure 1P–T).

**Figure 2:**
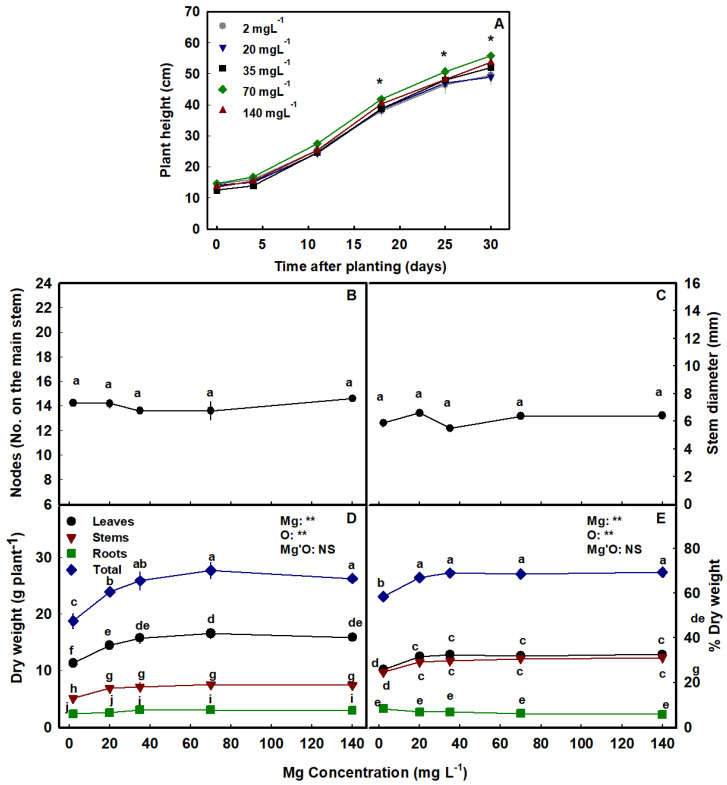
Effect of Mg supply on development of cannabis plants. Plant height (**A**), number of nodes on the main stem (**B**), stem diameter (**C**), dry weight (**D**), and % dry weight (**E**) of leaves, stems, roots, and total plant biomass. Data are the means ± SD (*n* = 5). Asterisks (in **A**) and different letters (in **B**–**E**) represent significant differences between Mg treatments by Tukey’s HSD test (α = 0.05). Results of a two-way ANOVA (in **D**,**E**) are indicated as: **, *p <* 0.05; NS, not significant; *p >* 0.05; O is organ, and Mg'O represents the interaction between Mg and O. Where not seen, error bars are smaller than the symbol size.

The Mg treatments affected the development of the plants. Plant height at the termination of the experiment was significantly lower in plants that received 2–20 mg L^−1^ Mg than in the 70–140 mg L^−1^ plants, but the effects were small with differences between treatments ~6 cm. Plant biomass was lowest in plants that received 2 mg L^−1^ Mg compared to the other treatments, a slight significant reduction in plant biomass was observed also under 20 mg L^−1^ Mg (Figure 2A,D), and there were no significant differences in plant biomass between plants that received 35–140 mg L^−1^ Mg (Figure 2D). The percentage of dry weight of the aerial plant organs was similarly affected and was lower in the low Mg treatment than in all other treatments, which showed no effect (Figure 2E). The level of Mg supplied to the plants did not affect the number of nodes on the main stem nor stem diameter (Figure 2B–D).

### 3.2. Physiological Parameters

**Figure 3:**
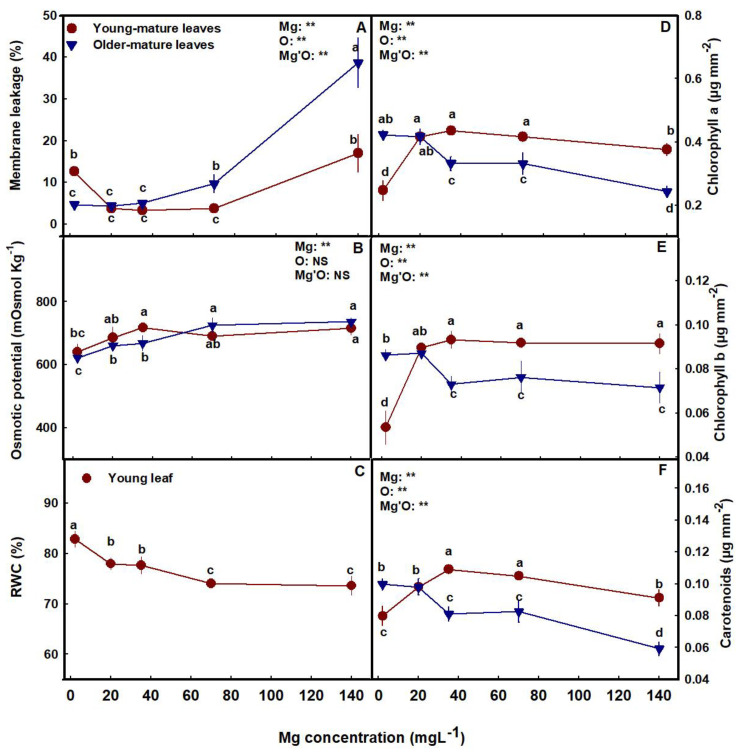
Effect of Mg supply on physiological characteristics of medical cannabis plants. Membrane leakage (**A**), osmotic potential (**B**), relative water content (RWC) (**C**), chlorophyll a (**D**), chlorophyll b (**E**), and carotenoids (**F**) in young-mature and older-mature leaves. Data are the average ± SD (*n* = 5). Different letters represent significant differences between treatments by Tukey’s HSD test at α = 0.05. Results of a two-way ANOVA are indicated as ** *p* < 0.05; NS, not significant; *p* > 0.05; O is organ, and Mg'O represents the interaction between Mg and O. RWC is presented for young-mature leaves.

Membrane leakage, an indicator of tissue stress, was lower in the young-mature leaves under the range of 20–70 mg L^−1^ Mg than under a lower or high Mg supply. This demonstrates sensitivity of the plant tissues to Mg deficiencies (2 mg L^−1^ Mg) and toxicity (140 mg L^−1^ Mg) in young-mature leaves. However, in older-mature leaves, sensitivity was observed only under high concentrations (70–140 mg L^−1^ Mg) (Figure 3A). This is in accord with the visual appearance of toxicity and deficiency reported above (Figure 1N–O).

Osmotic potential increased with the increase of Mg supply from deficient to optimal supply in both young-mature and older-mature leaves (Figure 3B), and in young-mature leaves, RWC decreased with the increase of Mg (Figure 3C).

The concentrations of the photosynthetic pigments tested, chlorophyll a, chlorophyll b, and carotenoids differed between the young-mature and the older-mature leaves and were affected by the Mg treatments (Figure 3D–F). In young-mature leaves, the concentrations were lowest at the deficiency treatments (2–20 mg L^−1^ Mg) and increased with Mg supply up to 20 or 35 mg L^−1^ (Figure 3D–F). The older mature leaves showed an opposite trend of a reduction in pigment concentrations with the increase in Mg supply, resulting in highest pigment concentrations at the lowest Mg treatments (2–20 mg L^−1^ Mg).

**Figure 4:**
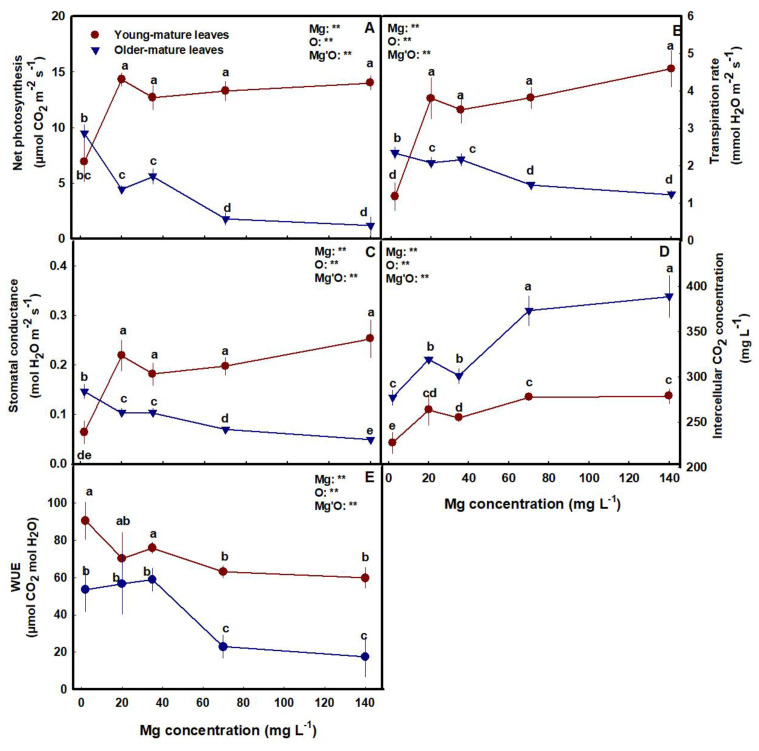
Effect of Mg supply on gas exchange parameters in young-mature and older-mature leaves. Net photosynthesis rate (**A**), transpiration rate (**B**), stomatal conductance (**C**), intercellular CO_2_ concentration (**D**) and WUE (**E**). Presented data are the average ± SD (*n* = 5). Different letters above the means represent significant differences between treatments by Tukey’s HSD test at α = 0.05. Results of a two-way ANOVA are indicated as ** *p* < 0.05; O is organ, and Mg'O represents the interaction between Mg and O.

Mg supply affected photosynthesis and gas exchange parameters in both young-mature and the older-mature leaves (Figure 4A–E). In the young-mature leaves, photosynthesis rate, transpiration rate, stomatal conduction, and internal CO_2_ concentrations were lowest under limited Mg supply (2 mg L^−1^) and increased with Mg supply up to the concentrations of 20 mg L^−1^. There were generally no differences in photosynthesis and gas exchange parameters between plants that received 70–140 mg L^−1^ Mg. In the older-mature leaves, the opposite trend was observed. Photosynthesis rate, transpiration rate, and stomatal conduction were highest under the low Mg supply (2 mg L^−1^), and decreased with an increase in the Mg supply. Internal CO_2_ concentrations showed a similar trend to young-mature leaves, however, the concentrations in the older-mature leaves were higher than in the young-mature leaves. Water use efficiency (WUE) was highest under low Mg supply and decreased with the increase in Mg levels above 35 mg L^−1^ in both leaves, and was higher in the young-mature leaves than the older leaves.

### 3.3. Macro- and Micronutrient Concentrations

**Figure 5:**
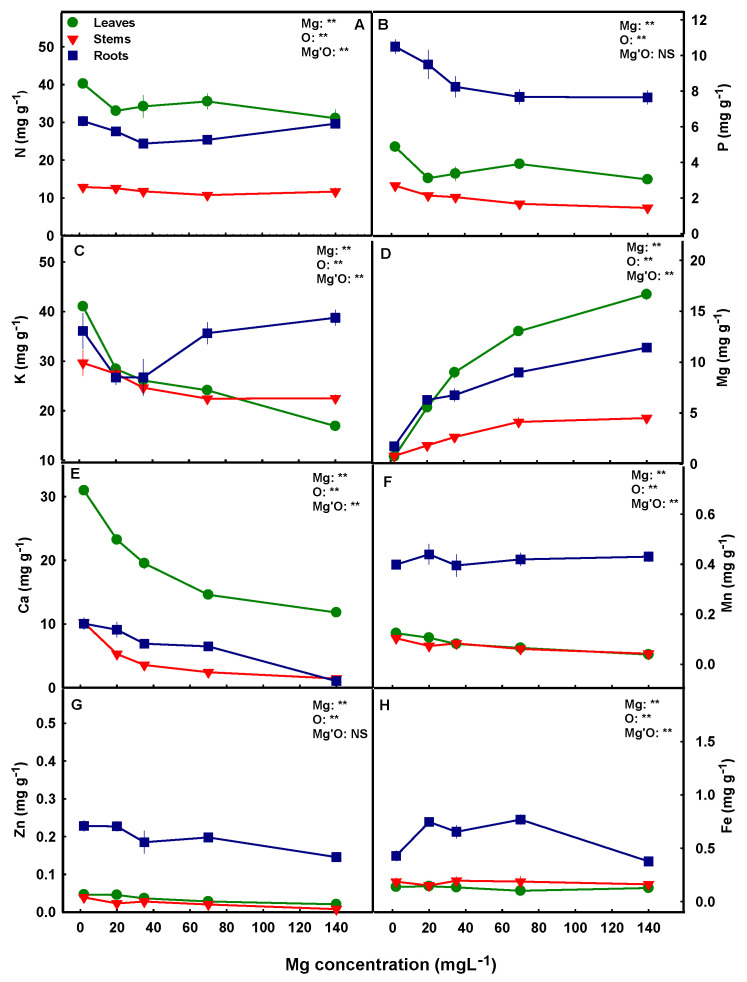
Effect of Mg supply on macro- and micronutrient concentrations in leaves, stems, and roots of medical cannabis plants. Total N (**A**), P (**B**), K (**C**), Mg (**D**), Ca (**E**), Mn (**F**) Zn (**G**), Fe (**H**). Presented data are the average ± SD (*n* = 5). Results of a two-way ANOVA indicated as ** *p* < 0.05; NS, not significant; *p* > 0.05; O is organ, and Mg'O represents the interaction between Mg and O.

Mg supply affected the uptake of Mg into the root and its translocation to the shoot, as can be seen by the general increase in Mg concentrations in the roots, leaves, and stems with the increase in Mg supply throughout the concentration range tested (Figure 5D). Ca uptake and translocation showed an opposite trend. Under the lowest Mg supply (2 mg L^−1^), Ca accumulated to the highest concentrations in all plant organs, and it decreased with the increase in Mg supply up to the concentration of 140 mg L^−1^, suggesting competition for uptake between the two cations (Figure 5E). Higher Ca concentration was observed in leaves compared to stems and roots (Figure 5E). K accumulation as well was highest in the shoot organs under the lowest Mg supply (2 mg L^−1^) (Figure 5C), and it decreased with the increase in Mg concentration up to the concentration of 70 or 140 mg L^−1^. Unlike leaves and stems, K concentration in the root increased with the increase in Mg supply above 35 mg L^−1^, which indicates accumulation of the cation in the root under high Mg concentrations (Figure 5C). N and P concentrations in the plant organs were affected as well by Mg supply. The concentrations of both nutrients were highest under Mg deficiency concentrations (Figure 5A,B), with N presenting a high level of shoot accumulation, in contrast to P which showed higher accumulation in the root.

**Figure 6:**
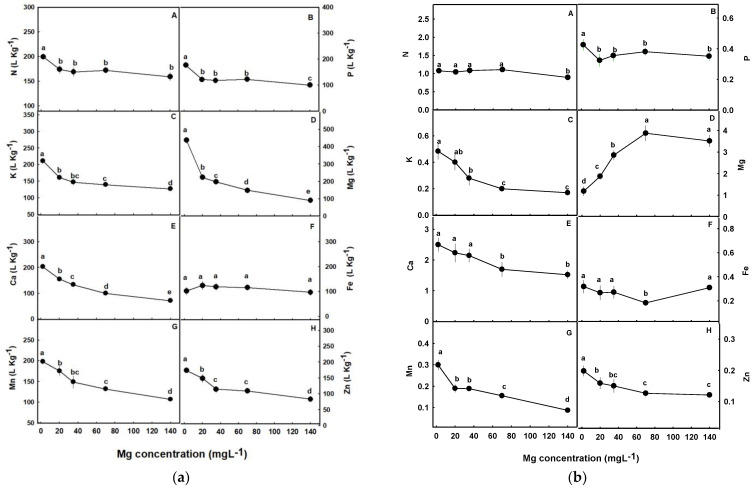
Effect of the Mg supply on bioaccumulation coefficients (BC) (**a**), and translocation factors (TFs) (**b**) of macro- and micronutrients in medical cannabis plants. Data are means ± SD (*n* = 5). Different letters above the means represent significant differences by Tukey’s HSD test (α = 0.05).

To evaluate whether the effect of Mg on accumulation of the minerals in the plant tissues was dominated by root uptake, *in-planta* translocation, or by an interaction of the two processes, a translocation factor (TF) and the bioaccumulation coefficient (BC) were calculated (Figure 6a,b). A common trend is apparent for the uptake of the macronutrients N, P, K, Mg and Ca under limited Mg supply. The BC analysis demonstrates that uptake of these mineral nutrients was highest under the deficiency concentration (2 mg L^−1^) than in all other treatments tested (Figure 6a(A–E)). With further increase of Mg supply, the uptake of Mg, Ca, and K decreased throughout the concentration range tested (Figure 6a(C–E)).

P uptake was steady throughout the concertation range of 20–70 mg Mg L^−1^ but reduced under higher supply (140 mg L^−1^, Figure 6a(B)), while N uptake was steady from 20–140 mg Mg L^−1^ (Figure 6a(A)). The TF analysis reveals that the root-to-shoot translocation of Mg increases with the increase in Mg supply up to the concentration of 70 mg L^−1^, K and Ca translocation decreased, P showed a high translocation to the shoot at the lowest Mg treatments (2 mg L^−1^) than in all other treatments, while N translocation was lowest under the Mg supply level of 140 mg L^−1^ (Figure 6b(A–E)).

Accumulation and uptake of micronutrients were affected as well by the level of Mg supplied to the plants. We have identified higher concentrations in the roots than in the shoot organs for all micronutrients tested (Fe, Zn, Mn,) (Figure 5F–H). Zn and Mn concentrations decreased with the increase in Mg supply in all plant organs, except root’s Mn which was not affected by the Mg treatments (Figure 5F–G). Fe concentration in the leaves and stems were not affected by the level of Mg supplied to the plants, however in the roots, the concentration was highest under the concentration range of 20–70 mg L^−1^ Mg than under lower or higher Mg levels (Figure 5H). The BC and TF analyses revealed that uptake as well as root-to-shoot translocation of Mn and Zn decreased with the increase in Mg supply (Figure 6a(G–H),b(G–H)), but no significant effects were found for Fe, except for a small possibly random reduction in the TF under 70 mg L^−1^ Mg (Figure 6a(F),b(F)).

## 4. Discussion

Minerals are major determinants of plant growth, function, and fertility in nature and agriculture [11]. Essential nutrients are known to have structural roles in the plant, and to involve in plant function also by a range of regulatory mechanisms [36]. Magnesium is one of the essential nutrients for plants. It plays a key role in photosynthesis, and is known to activate more than 300 enzymes thus participating in a range of key processes in the plant [24,37,38]. Similar to other nutrients, a defined supply level of Mg is required for optimal plant development and function, and higher or lower application rates result in deficiency or toxicity damages [39].

The present study was designed to investigate the impact of Mg nutrition on drug-type (medical) cannabis plants at the vegetative phase of growth, with the goal to increase our understanding of the nutritional requirements of the plants, and towards the development of an optimal fertigation regime. The results confirmed the hypothesis that Mg nutrition affects the plant’s morpho-physiology, and elicits developmental changes associated with changes to the plant ionome. Our major findings are that growth and function of the cannabis plant are optimal under Mg supply of 35–70 mg L^−1^, and impaired under the lower-deficient Mg input of 2 mg L^−1^. Supply of 20 mg L^−1^ resulted in but a small reduction in growth compared to the optimal supply range. The highest Mg level of 140 mg L^−1^ had a small inhibiting effect on plant function (gas-exchange parameters and pigment contents, especially in old leaves), but it did not inhibit significantly morphological development and biomass accumulation. Taken together, the findings suggest 35–70 mg L^−1^ Mg as the optimal concentration range for cannabis plant development and function. These results add understanding concerning effects of the macronutrient Mg, to the information available about effects of the three macronutrients N, P, and K on medical cannabis plants at the vegetative growth phase [8,13,14], and the reproductive growth phase [7,15,16,18,40,41,42].

### 4.1. Plant Visual Appearance, Development, and Function

A plant’s sufficiency range for a particular essential nutrient is defined as the concentration range required to meet the nutritional need of the plant for maximum growth. This range varies between plant species and nutrients. Nutrient levels higher or lower than the plant’s sufficiency range reduce plant growth and health due to deficiency or toxicity damages, and can impose a visual response in the plant [7,43]. The visual symptoms induced by deficiency and toxicity are often used as an initial indicative tool of deficiency and toxicity conditions. However, by the time such symptoms appear, plant function and productivity may have already been considerably compromised [43,44].

Magnesium is required by the plant in large quantities, therefore insufficient Mg levels are not uncommon in production systems [45,46]. Mg toxicity is less common and is known to occur when plants are exposed to low Ca and high Mg conditions, such as under cultivation in serpentine soil [47]. Visual symptoms of severe Mg deficiency in plants first appear as leaf yellowing in the form of interveinal chlorosis on older and mature leaves [48,49]. In the present study, we report similar trends for the appearance of visual deficiency symptoms in cannabis. Plants that received a deficient Mg supply of 2 mg L^−1^ first developed Mg-deficiency symptoms in old leaves at the bottom of the plant that under prolonged deficiency progressed towards younger-mature leaves (Figure 1K,F). This visual deficiency trend is known to result from Mg ability for storage and translocation in the plant; as Mg can be stored in large quantities in vacuoles and serve as a pool for maintaining Mg homeostasis in other cells. Owing to its high mobility in the plant, older plant organs serve as Mg pools, supplying Mg for younger, growing organs that have a high demand for Mg [50]. Furthermore, Mg deficiency elicits degradation of Mg-containing constituents in old-mature leaves, such as chlorophyll, hence increasing the availability of unbound Mg for retranslocation to younger tissue [51]. Thereby, when Mg supply is limited, its translocation from mature to young developing leaves is enhanced, and this preferential remobilization results in the initial and more severe appearance of deficiency symptoms in old leaves [10,47]. The deficiency symptoms were not apparent under the supply levels of 20–140 mg L^−1^ Mg, although plant biomass was compromised slightly also under 20 mg L^−1^ Mg. This demonstrates that visual symptoms of Mg deficiency in cannabis should not be utilized as a first-line diagnostic tool of the nutritional status of the plants since they appear only under severe deficiency.

The limited availability of Mg in young-mature leaves under 2 mg L^−1^ Mg input that was apparent by the visual deficiency symptoms, resulted in reduction of the Mg-containing photosynthetic pigments chlorophyll a and chlorophyll b, as well as carotenoids in the leaf (Figure 3). A decrease in these pigments’ concentrations in response to Mg deficiency was reported for numerous crops [52,53,54], and was accompanied by a reduction of the photosynthesis rate (Figure 4A). Such a reduction results from the major roles that Mg have in photosynthesis and related processes in the chloroplast [48]. It is required for maintaining the chloroplast ultrastructure, i.e., thylakoid stacking and grana formation [55], for chlorophyll synthesis [56], and for activation/activity of key enzymes involved in photosynthetic CO_2_ fixation including Rubisco [57]. It is therefore not surprising that the deficient availability of Mg imposed a reduction of net CO_2_ assimilation rate in the cannabis plants (Figure 4), as was reported for many plant species [58,59,60,61]. The reduction of photosynthesis under Mg deficiency is attributed also to a feedback inhibition by excessive accumulation of sugars in young-mature leaves. It has long been recognized that the rate of photosynthesis and expression of photosynthesis genes are negatively affected by sugar accumulation [19,62] and that Mg- deficiency induces elevation of sugar concentration in leaves. The elevation of sugar concentration is known to result from depression of sucrose transport from young-mature source leaves under Mg deficiency [63,64], due to impaired phloem loading likely by the sucrose/H^+^ symporter BvSUT1, which requires Mg-ATPase to generate H^+^-motive force [65].

The observed increase in membrane leakage at the young-mature leaves under Mg-deficient conditions (Figure 3A), which reflects oxidative damage to the cell membranes [34], likely reflects increased oxidative stress in these leaves. Over-production of reactive oxygen species (ROS), and oxidative damage to Mg-deficient leaf tissue is well documented [66]. It is considered to result from the restricted CO_2_ fixation under Mg deficiency due to reduced biochemical utilization of the absorbed light energy, and thereby induced increased formation of ROS. The increased concentration of ROS induces subsequent degradation of chlorophyll [67]. Accordingly, differences between plants in the extent of visual symptoms of Mg deficiency in leaves was suggested to be related to differences in the production or detoxification of ROS in the chloroplasts [68].

The effect of the Mg treatments on the rate of photosynthesis in young-mature vs. old-mature cannabis leaves, is in accord with the effects we identified on the concentrations of the photosynthetic pigments, which were lowest in the young-mature leaves under the 2 mg L^−1^ Mg supply (Figure 3D–F). Photosynthesis of old leaves was not reduced by Mg deficiency, likely due to the lack of effect on the concentration of the pigments, which were highest in these leaves under Mg deficiency (Figure 3D–F and Figure 4A). The lesser impact of the Mg-deficiency stress on older mature leaves is apparent also by the lack of impact on membrane leakage, while under higher Mg concentrations, photosynthesis was reduced in accord with the increase in tissue damage that can be related to leaf aging (Figure 3A). Interestingly, these results demonstrate a preservation of leaf gas exchange activity in aging leaves under Mg-deficiency, unlike reports for some other plants that identified stimulation of senescence by Mg-starvation [69].

Stomatal conductance, and consequently the transpiration rate and internal CO_2_ concentration were lowest in the young-mature leaves under the deficient 2 mg L^−1^ Mg supply, but not in older mature leaves (Figure 4). Such a specific restriction of transpiration in some source leaves by Mg starvation was reported for other plant species [61,69]. Kobayashi and Tanoi [70] suggested that such specificity results from stomatal closure by generation of ROS at defined stages of the deficiency stress, since ROS is known as a second messenger for stomata closure in several hormonal signaling [71]. This notion is supported by our results, as reduced stomata conductance in the young-mature leaves under the severe Mg-deficiency was accompanied by increased membrane leakage (Figure 3A), which is considered to result from increased lipid peroxidation by ROS, but was not identified in older mature leaves. The lower stomatal conductance under the Mg-deficiency conditions in the young-mature leaves was manifested as expected also by reduced transpiration and osmotic potential and increased relative water content (Figure 3 and Figure 4). Recent results suggest that Mg is involved in regulation of stomatal opening also via a tonoplast-localized Mg transporter in the guard cells [72].

Mg deficiency is well documented to inhibit plant growth and development in a variety of species [26], including spinach [73], radish [74], sugar beet [75], and citrus [76]. We report here that the cannabis plants responded similarly to Mg scarcity, and the imposed morphological constraints were in accord with known responses for other crops, demonstrating suppression of shoot development. Specifically, plant height and total shoot biomass were restricted under low Mg supply (2–20 mg L^−1^ Mg) (Figure 2) although the restriction under 20 mg L^−1^ Mg was small. The suppressed biomass production and morphological development under 2–20 mg L^−1^ Mg suggest this to be a deficiency concentration range for growth and development at the vegetative stage of growth.

Our morphological testing demonstrated that the cannabis root is less sensitive to Mg supply then the shoot. The lowest supply rates of 2 mg L^−1^ that suppressed shoot development by 33.5% compared to the optimal 35 mg L^−1^ Mg, reduced root biomass accumulation by only 23.7% (Figure 2D). These results are in accord with previous studies for other plants [54,64] that demonstrated as well that roots are less affected than the shoot by Mg deficiency. This is supported also by transcriptome profiling in Arabidopsis [28], that identified that under Mg starvation fewer genes were differentially regulated in roots than in leaves, and root development was less affected than the shoot. Furthermore, a proteomics study of the root hair in maize showed an upregulation of many ribosomal proteins under Mg deficiency [77], indicating an impact also on protein synthesis in root hair, pointing at a potential function of the root in plant response to Mg deficiency. The relative sensitivity of the root vs. shoot to Mg deficiency can differ between species and growing conditions, and reports for some crops demonstrated higher or equal sensitivity of the shoot vs. the root to Mg-limitations [50].

### 4.2. Interrelations between Mg Supply and the Cannabis Plant Ionome

As a macronutrient that is utilized by the plant in large quantities, the availability of Mg in the plant organs should be regulated to satisfy the considerable requirements for optimal plant function. To take up and maintain sufficient concentrations of Mg, plants have evolved efficient systems for Mg uptake, storage, and translocation [19]. Our results that show an increase in Mg concentrations in all plant organs with the increase in Mg supply, reflect stimulation of Mg uptake in cannabis upon exposure of the plant roots to increasing Mg dosages. The enhanced accumulation in the shoot was facilitated by an increase in root-shoot translocation, as is evident by the concomitant increase in the translocation factor (Figure 6b(D)). The increase in Mg accumulation in the shoot under increased Mg supply was considerable—to the point that the concentration in the leaves under 140 mg L^−1^ supply reached 195% of the optimal concentration found under a 35 mg L^−1^ input (Figure 5D). Such over-accumulation of Mg in shoot organs is known to occur in other plants as well [78] and was previously suggested to act as an *in-planta* reservoir by compartmentation of Mg in the vacuoles, which also acts as a tolerance mechanism preventing cellular exposure to damaging high concentrations [70].

Plant development and function can be inhibited severely if Mg becomes limiting under restricted supply. Root systems show quick responses to Mg depletion by boosting the uptake of Mg [45,79,80]. Several MGT/MRS2 family members in Arabidopsis (AtMGT6, AtMGT7), rice (OsMGT1), and maize (ZmMGT10) were shown to be responsible for elevating Mg uptake to maintain Mg homeostasis under Mg deficiency conditions [47]. Our results for cannabis show as well a considerable enhancement of plant Mg uptake under restricted Mg supply, as the bioaccumulation factor increased with the reduction in Mg input throughout the concentration range tested.

Magnesium is known for its interactions with other plant minerals for root uptake [37], which may result in changes to ion transport and concentrations in the plant. The increased concentration of Mg in the root growing solution increases the total cation concentration in the solution and thereby the competition between the positively charged cations for root uptake. Especially documented are the antagonistic effects of Mg in the root solution on root uptake of Ca and K [81]. Our nutritional analysis revealed that in cannabis plants as well, elevated Mg inputs restricted Ca uptake into the plants (Figure 5E). Ca uptake was highest under Mg deficiency (2 mg L^−1^), as can be seen by the highest bioaccumulation coefficient and its reduction with the increase in Mg supply throughout the concentration range tested (Figure 6a(E)). Elevated Ca in the plant in response to Mg deficiency is well documented and was reported for many plants [30,75,82].

In addition to Mg effects on Ca uptake, Ca translocation to the shoot was impaired as well with the increase in Mg supply. This is demonstrated by the reduction in the translocation factor under supply concentrations >20 mg L^−1^ (Figure 6b(E)), and by the decrease in Ca concentration in all plant organs with the increase in Mg supply throughout the concentration range tested (Figure 5E). Mg deficiency significantly increased Ca concentration in the leaves; a reduction of 5.68 mM in leaves’ Mg between the highest and the lowest Mg treatments in our study (2–140 mg L^−1^ Mg) resulted in 61% increase in leaves’ Ca concentration. These results are in accord with reports for other crops [83,84] which identified as well a decrease in Ca in response to an increase in Mg supply. Taken together, our results demonstrate that the Mg-induced reduction in Ca concentration in the cannabis shoot is an integrated outcome of inhibition of Ca uptake and root-shoot translocation of Ca.

Calcium is well known to play a fundamental role in membrane stability and cell integrity, and Ca deficiency induces membrane damage that increases the leakage of solutes from cells [10]. In the cannabis plants that received high concentrations of Mg, leakage of solutes from the tissue increased (Figure 3A), which suggests that Ca-deficiency effects play a role in the cannabis plant response to high Mg levels.

The effect of Mg supply on Ca nutrition is similar to the effect on K. Uptake and translocation of K were inhibited as well by high Mg supply. K uptake was highest under Mg deficiency, as was observed for several plant species [30,75] and its translocation to the shoot decreased with the increase in Mg supply up to 140 mg L^−1^. Similar trends were found in maize, onion, and rice [61,83,85]. Unlike leaves and stems, K concentration in roots increased under high Mg supply (Figure 5C), which implies that high Mg concentrations were more restrictive for K translocation to the shoot then for K uptake into the roots.

There is much evidence for a strong antagonistic effect of K on Mg uptake [13,38], but reports on the influence of Mg on K uptake are scarce and contradictory [30,86]. In cannabis, the calculated bioaccumulation coefficient demonstrates that total K uptake into the plant decreased with the increase in Mg supply (Figure 6a(C)). The effects of Mg on root K uptake is considered to result from effects on the active component of K uptake across the root plasma membrane, which depends on ATPase activity that is disrupted under Mg deficiency [38]. Interestingly, despite a high uptake of K into the root, there was an inhibition of its translocation to the shoot, causing the roots' K to increase considerably under high Mg supply (Figure 5C). This demonstrates restriction of root-to-shoot transport of K under high Mg supply, as is evident also by the decrease in the translocation factor (Figure 6b(C,E)). The effect of Mg on the uptake and root-to-shoot translocation of Ca and K, and the significant decrease in their concentrations in the plant has been documented in several species [86,87,88]. The observed competitive interaction between Mg, Ca, and K for uptake and translocation in the plant is related to uptake systems specificity, such as the non-selective ion channel found in both leaves and roots that can transport Ca and K, vs. the high-affinity H/Mg exchanger that is expressed under Mg depletion conditions [89].

A noteworthy trend identified by the nutritional analysis is that low Mg supply stimulates accumulation of some minerals (N, P, K, Ca, and Zn) in the plant, while accumulation of other nutrients (i.e., Fe) was not affected (Figure 6a). This preferential accumulation suggests specificity in the response of ion uptake mechanisms to Mg supply. Reduced concentration of specific ions in the root growing solution can affect the uptake of other nutrient-ions by competition for uptake or by charge-balance aspects. Mg is well known to compete with other cations, including NH_4_, Ca, K, and Mn for root-uptake [32], and enhanced Mg supply suppresses cation uptake and stimulates anion uptake [90]; this was apparent also in the present study as the concentrations of these nutrients and their bioaccumulation factors reduced with the increase of Mg supply over 2 mg L^−1^ (Figure 5 and Figure 6a). The stimulated uptake of the nutrients under Mg deficiency did not compensate for the limited Mg supply, as plant growth was compromised under the limited Mg availability (Figure 2).

The highest accumulation of N in the cannabis plants was found in the leaves (Figure 5A). This is consistent with previous studies on cannabis from our laboratory [7,14], and was also reported for other crops [91,92,93]. This distribution pattern supports the high requirements of the leaves for nutrients, which during rapid vegetative growth and high metabolism are the highest-demanding organ for nutrients. Both N and Mg are structural components of chlorophyll molecules; Mg is found in the center of the porphyrin ring in the chlorophyll where it coordinates with four N atoms [94]. Deficiency of either N or Mg therefore leads to chlorophyll reduction, and ultimately affects energy transfer in the photosystems [95], which ultimately restricts plant growth. Some studies identified a suppression of root N uptake under Mg deficiency and suggested that the symptoms caused by Mg deficiency might be partially derived from N deficiency [96]. Our results uncovered that in the cannabis plant, Mg-deficiency does not suppress N uptake, as the bioaccumulation factor and N concentrations in the leaves and roots were highest under Mg deficiency (Figure 5A and Figure 6a(A)). Therefore, we conclude that N limitation is not a factor in the Mg-deficiency physiological responses in cannabis.

Unlike N, the highest concentration of P in the cannabis plants was found in the roots (Figure 5B). This is in accord with our prior results for two other cannabis genotypes at the vegetative growth phase [8], but is unlike the distribution of P at the reproductive growth phase that favored accumulation in the reproductive (inflorescence) organs [7]. Root accumulation of P is considered to be a defense mechanism against P toxicity in the shoot [10]. It is accompanied by sub-cellular compartmentation in the vacuole which prevents the accumulation of toxic levels of P in the cytosol. Under optimal P nutrition, ~70–95% of the intercellular Pi was reported to compartmentalize in the vacuoles, thus transporter regulation under various nutritional conditions maintains Pi cellular homeostasis [97].

The accumulation of P in the plant was highest under Mg deficiency, and both P concentrations and the bioaccumulation factor were highest under the 2 mg L^−1^ deficiency treatment (Figure 5B and Figure 6a(B)). However, stimulation of P accumulation under limited-Mg that was reported also for soybean [98] is not a uniform response in plants, as other studies reported synergism between Mg and P uptake, i.e., stimulated P uptake under increased Mg availability [99]. The resulting co-limitation of Mg and P availability in the plant which was developed under Mg-limiting conditions was suggested as a mechanism for restriction of plant production under Mg deficiency [99]. Our results that show stimulation rather than inhibition of P uptake under limited Mg availability demonstrate that in cannabis, P limitation is not a restrictive factor under Mg deficiency.

Micronutrients play an eminent role in plant growth, development, and metabolism and have considerable effects on physiological functions of plants [100,101]. They are known to interact with other minerals, and such interactions can cause deficiency or toxicity damages in plants [32]. Very little information is available about micronutrient nutrition in the cannabis plant, and it was therefore important to dissect the effect of Mg on uptake and translocation of micronutrients and in relation to plant function. Our results uncovered considerable effects of Mg supply on micronutrient concentrations in the cannabis plant organs and identified characteristic translocation and bioaccumulation trends. Most Fe, Mn, and Zn accumulated in the roots (Figure 5F–H), pointing at a compartmentation strategy for prevention of excess concentrations in shoot tissues, or for storage [14]. The translocation factors for the three micronutrients were accordingly <1, which represents root localization. This supports results from our previous studies that showed as well preferential accumulation of these micronutrients in the cannabis roots.

Mg nutrition had a considerable effect on Mn uptake into the plant and its translocation to the shoot. Mn concentrations in leaves and stems decreased with the increase in Mg supply up to 140 mg L^−1^, as can be seen by the decrease of the translocation factor (Figure 6a(G)), while root’s Mn was not affected by the Mg treatments. Despite the lack of effect of Mg on Mn in the root, the total uptake of Mn was restricted by increasing Mg levels, as is demonstrated by the bioaccumulation coefficient (Figure 6a(G)). This suppression of both uptake and root-to-shoot translocation of Mn relates to the well-known competition between the two divalent cations for membrane transport. This antagonistic interrelation source from cations competition for apoplastic binding sites and for membrane uptake, for example through nonselective cation channels, which can be utilized by both monovalent and divalent cations [102]. This competition for uptake is adventitious to the plant under conditions of Mn oversupply, and an increase in Mg was indeed reported to alleviate Mn toxicity in a range of crops [61,103,104,105,106].

The critical toxicity concentration of Mn varies widely among plant species and environmental conditions and ranges from 200 to 5300 mg Mn kg^−1^ [100]. In our Mg-deficient cannabis plants, most Mn accumulated in the roots and reached up to 425 mg Mn kg^−1^ (on average throughout the Mg concentrations range tested). Thus, the cannabis plants grown under the severe Mg deficiency in our study might have suffered Mn toxicity at the roots. Information about cannabis response to Mn dosages is required to determine if root Mn toxicity plays a role in the plant’s response to Mg deficiency. Mn is highly mobile in the plant and is easily transported in the xylem from roots to the shoot [107]. However, restriction of xylem transport might have been imposed by the strong depression of transpiration in young-mature leaves under Mg deficiency (Figure 4B), which might have further contributed to Mn root accumulation. However, this is unlikely since in spite of the restriction of transpiration under severe Mg-deficiency, Mn as well as other cations (P, K, Ca, Zn) accumulated to the highest concentrations at this treatment.

The same trend was observed for Zn accumulation in response to different Mg levels. Zn uptake was highest under Mg deficiency (Figure 6a(H)) resulting in high Zn concentrations in all plant organs (Figure 5G). Zn has a similar ion radius to Mg [100], which could have facilitated the high absorption of Zn under Mg deficiency (2 mg L^−1^ Mg). Enhancement of Zn uptake under Mg deficiency was reported for several plant species [53,108,109], and so was the reciprocal inhibition of Mg uptake by high Zn concentrations [110,111]. This supports the impact of the cation-cation antagonistic interactions for uptake of these two cations [112]. Mg nutrition also affected Zn translocation from root-to-shoot in the cannabis plants that was highest under low Mg supply and decreased with the increase in Mg supply up to 70 mg L^−1^ (Figure 6b(H)). The enhancement of Zn translocation under Mg deficiency can be related to activity of an Mg/H exchanger in the vascular system, that acts at low Mg concentrations and was reported to transport also Zn [26,113]. Taken together, our results identified a considerable effect of Mg on uptake and translocation of the micronutrients Mn and Zn in the cannabis plants, and demonstrated a preferential higher accumulation of Mn, Zn, and Fe in the roots compared to the shoot organs.

## 5. Conclusions

Results of the current study revealed that Mg nutrition has a considerable impact on the morpho-physiological function of the cannabis plant at the vegetative growth phase, which are associated with changes to the ionome. The concentration of 2 mg L^−1^ Mg supply was identified to be a deficiency concentration resulting in: (i) Visual deficiency symptoms, (ii) Low morpho-physiological function, and (iii) Reduced total plant biomass (compared to the supply level of 35 mg L^−1^ Mg). Under higher supply levels of Mg, 35–70 mg L^−1^, plant growth was similar and higher then under the deficiency level, although some reduction in plant function was apparent in old-mature leaves of the 70 mg L^−1^ treatment. The highest supply level of 140 mg L^−1^ Mg, resulted in visual toxicity symptoms only in the old leaves at the bottom of the plants, and a slight decrease in physiological function, which did not affect biomass accumulation in the plants. Taken together, the results reveal that 35–70 mg L^−1^ Mg is the optimal concentration range for cannabis plant development at the vegetative growth phase. To avoid unnecessary financial expenses and environmental consequences caused by excessive use of fertilizers, 35 mg L^−1^ Mg can be applied in commercial production, as it is sufficient for excelled morpho-physiological function in the medical cannabis cultivar tested. 

## Data Availability

All data are included in the manuscript.
